# The Effect of Exogenous Sex Steroids on the Vaginal Microbiota: A Systematic Review

**DOI:** 10.3389/fcimb.2021.732423

**Published:** 2021-11-12

**Authors:** Larissa K. Ratten, Erica L. Plummer, Catriona S. Bradshaw, Christopher K. Fairley, Gerald L. Murray, Suzanne M. Garland, Deborah Bateson, Gilda Tachedjian, Lindi Masson, Lenka A. Vodstrcil

**Affiliations:** ^1^ Central Clinical School, Monash University, Carlton, VIC, Australia; ^2^ Melbourne Sexual Health Centre, Alfred Health, Carlton, VIC, Australia; ^3^ Melbourne School of Population and Global Health, The University of Melbourne, Parkville, VIC, Australia; ^4^ Department of Obstetrics and Gynaecology, The University of Melbourne, Parkville, VIC, Australia; ^5^ Centre for Women’s Infectious Diseases Research, The Royal Women’s Hospital, Parkville, VIC, Australia; ^6^ Infection and Immunity, Murdoch Children’s Research Institute, Parkville, VIC, Australia; ^7^ Family Planning NSW, Sydney, NSW, Australia; ^8^ Discipline of Obstetrics, Gynaecology and Neonatology, Faculty of Medicine and Health, The University of Sydney, Sydney, NSW, Australia; ^9^ Life Sciences Discipline, Burnet Institute, Melbourne, VIC, Australia; ^10^ Department of Microbiology, Monash University, Clayton, VIC, Australia; ^11^ Department of Microbiology and Immunology, Peter Doherty Institute for Infection and Immunity, University of Melbourne, Melbourne, VIC, Australia; ^12^ Institute of Infectious Disease and Molecular Medicine and Department of Pathology, University of Cape Town, Cape Town, South Africa; ^13^ Centre for the AIDS Programme of Research in South Africa, Durban, South Africa

**Keywords:** vaginal microbiota, hormonal contraceptives, menopausal hormone therapy, lactobacillus, Gardnerella vaginalis, oestrogen, progesterone, progestin

## Abstract

**Background:**

Exogenous sex steroids within hormonal contraception and menopausal hormone therapy (MHT) have been used for family planning and management of menopausal symptoms, without consideration of their effects on the vaginal microbiota. This is largely because their use predates our understanding of the importance of the vaginal microbiome on human health. We conducted a systematic review (PROSPERO: CRD42018107730) to determine the influence of exogenous sex steroids, stratified by oestrogen-containing or progestin-only types of contraception, and MHT on the vaginal microbiome, as measured by molecular methods.

**Methods:**

Embase, PubMed and Medline were searched for relevant literature published through to December 1st 2020. Eligible studies reported on the effect of specific exogenous sex steroids on the vaginal microbiome using a molecular method. Data regarding the ‘positive’, ‘negative’ or ‘neutral’ effect of each type of contraceptive or MHT on the vaginal microbiome was extracted and summarised. A positive effect reflected sex steroid exposure that was associated with increased abundance of lactobacilli, a change to, or maintenance of, an optimal vaginal microbiota composition, or a decrease in bacterial diversity (specifically reflecting a low-diversity optimal microbiota state), relative to the control group. An exogenous sex steroid was designated as having a negative effect on the vaginal microbiome if it resulted in opposing effects (i.e. loss of lactobacilli, a non-optimal microbiota state). When no significant change was found, this was considered neutral/inconclusive.

**Results:**

We identified 29 manuscripts reporting on the effect of exogenous sex steroids on the vaginal microbiome; 25 investigating hormonal contraceptives, and 4 investigating MHT. Oestrogen-containing contraception, particularly reflecting the combined oestrogen and progestin-containing contraceptive pill, had a positive effect on the composition of the vaginal microbiota. Progestin-only contraception, particularly reflecting depo-medroxyprogesterone acetate, had mixed effects on the microbiota. Among post-menopausal women using MHT, exogenous oestrogen applied topically was associated with increased prevalence of lactobacilli.

**Conclusion:**

Our findings suggest that oestrogen-containing compounds may promote an optimal vaginal microbiota, which could have clinical applications. The impact of progestin-only contraceptives on the vaginal microbiota is less clear; more data is needed to determine how progestin-only contraceptives contribute to adverse reproductive and sexual health outcomes.

## Introduction

Hormonal contraceptives have been in use since the 1960s and until recently, have not been examined for their influence on the vaginal microbiota. A vaginal microbiota associated with optimal reproductive and sexual health outcomes is characterised by *Lactobacillus* spp., although microbiome composition varies across geographical locations and specific populations (Fredricks et al., 2005; Ravel et al., 2011; Aldunate et al., 2013). Conversely, a non-optimal vaginal microbiota is typically characterised by reduced abundance of *Lactobacillus* spp. and is associated with negative sexual and reproductive health outcomes such as preterm delivery, miscarriage, low birthweight, pelvic inflammatory disease, and STI and HIV acquisition (Koumans et al., 1999; Wiesenfeld et al., 2002; Brotman et al., 2010; Chetwin et al., 2019; McKinnon et al., 2019). A non-optimal vaginal microbiota abundant in *Gardnerella* spp., including *G. vaginalis, Atopobium vaginae* and other facultative and strict anaerobes, is associated with the most common vaginal dysbiosis, bacterial vaginosis (BV) (Fredricks et al., 2005; Ravel et al., 2011; McKinnon et al., 2019). Prior systematic reviews and meta-analysis of observational data have shown that exogenous sex steroids, delivered as either oestrogen-containing and progestin-only contraceptives may exert a positive effect on the microbiota, with a decrease in BV (van de Wijgert et al., 2013; Vodstrcil et al., 2013). However, some progestin-only contraceptives (i.e. depo-medroxyprogesterone acetate [DMPA]), have been scrutinised for a suspected link between their use and an increased risk of HIV acquisition and transmission (Polis and Curtis, 2013; Deese et al., 2015; Dabee et al., 2021). Among post-menopausal women, the composition of the vaginal microbiota is implicated in vaginitis, which is a common condition in this population (Diem et al., 2018; The 2020 Genitourinary Syndrome of Menopause Position Statement of The North American Menopause Society, 2020). Vaginitis (formerly atrophic vaginitis) and other peri-and post-menopausal symptoms are managed with exogenous oestrogen within menopausal hormone therapy (MHT, also known as hormonal replacement therapy) in some cases (Castelo-Branco et al., 2005; Chollet et al., 2009). However, less is known about what constitutes an optimal vaginal microbiota in this population (Mitchell et al., 2017).

The use of hormonal contraceptives and MHT is rising globally (Contraceptive Use by Method 2019: Data Booklet. 2019) and clearly, these compounds need to be examined for their largely unappreciated effects on the vaginal microbiota. Advancements in molecular technology have allowed for more accurate and high-throughput studies examining the vaginal microbiota to be conducted. Given the uncertainty about the impact of specific hormonal contraceptives on the vaginal microbiota, this timely systematic review summarises the effect of specific oestrogen-containing or progestin-only contraceptives as well as MHT on the vaginal microbiota, and critically evaluates the strength of the findings.

## Methods

### Protocol and Registration

This systematic review was conducted in line with the PRISMA (Preferred Reporting Items for Systematic Reviews and Meta-analyses) statement (Page et al., 2021) and was prospectively registered with PROSPERO on the 5/09/2018 (International Prospective Register of Systematic Reviews; CRD42018107730).

### Search Process, Eligibility Criteria

We searched the electronic databases Embase, PubMed and Medline for relevant literature published through to December 1^st^ 2020 using the following strings: [(vagina* AND bacter* OR vagina*) AND micro* OR vagina*] AND flora AND [(hormon* AND contracept* OR oral) AND contracept* OR estrogen OR oestrogen OR progest*]. Studies were uploaded to Covidence (Veritas Health Innovation, Melbourne, Australia, www.covidence.org) and screened by two authors (L.K.R and E.L.P); with any disputes resolved by a third author (L.A.V). Conference abstracts were included for screening; only English language studies were considered and only data from human participants was examined.

To be eligible for inclusion, studies were required to report on the most commonly used sex steroids (i.e. oestrogen and/or progestin delivered as hormonal contraceptives or MHT) and provide a measure of the composition, stability and/or diversity of the vaginal microbiota by molecular methods (**Supplementary Table 1**). Molecular methods included i) quantitative polymerase chain reaction (qPCR), ii) 16S microarray, iii) next-generation sequencing and/or iv) Sanger sequencing. Studies were excluded if they 1) did not report on sex steroid use; 2) only measured the vaginal microbiota by non-molecular methods (such as culture and microscopy); 3) were qPCR studies that did not include at least one *Lactobacillus* target or one BV-associated bacteria; 4) only included pregnant women or women undergoing *in vitro* fertilisation; or 5) were reviews. Studies were not excluded based on trial design and could include cross-sectional studies, cohorts, and randomised trials. Where two studies presented data on the same population, only the most recent study was retained, unless one presented baseline cross-sectional data and the second presented longitudinal data, in which case both were included. Where studies measured the microbiota with molecular methods and reported sex steroid use but did not present findings on the effect of sex steroids on the microbiota, the study authors were contacted for additional details. Authors from seven studies were contacted *via* email, all responded and two provided additional data for three studies.

### Study Population and Interventions Assessed

We deliberately did not specify a population age, which resulted in two distinct study populations identified in the literature search; reproductive-aged women using hormonal contraception and post-menopausal women using MHT, which were analysed separately. In the reproductive-aged population, any kind of hormonal contraception type was included as a suitable intervention, however these predominantly reflected oestrogen-containing or progestin-only methods of hormonal contraception, as these are the most commonly used formulations. Where more than one sex steroid use was reported in the study population, all types were recorded. In the post-menopausal population, topical conjugated oestrogens were used. The comparator group varied between studies and included 1) baseline specimens prior to sex steroid initiation, women not using contraception, or MHT, or women using a non-hormonal contraceptive [i.e. copper IUD (Cu-IUD), condom use].

### Data Extraction

Data was extracted by two authors (L.R. and E.P.). Any discrepancies were resolved through discussion with a third author (L.V.). The following data were extracted: i) study location, ii) population characteristics (e.g. HIV status, sex-worker status, age, and ethnicity), iii) frequency of sampling, iv) molecular technique applied, v) molecular outcomes reported on, vi) comparator group used, vii) sex steroid source, viii) molecular findings and ix) summarised effect of sex steroid exposure (positive, neutral or negative).

### Data Analysis

For studies using next generation sequencing, the impact of exogenous sex steroids on vaginal microbiota was reported as relative abundance, bacterial diversity, community state type (CST)/vaginal microbiota cluster or group assignment, and change in these measurements over time during sex steroid exposure. All results were stratified by sex steroid type. Microarray and qPCR study findings were dependent on the panel targets tested. Most commonly, biologically significant *Lactobacillus* spp. (i.e. *L. crispatus, L. iners, L. gasseri, L. jensenii, L. johnsonii* and *L. vaginalis*) as well as BV-associated bacteria [i.e. *G. vaginalis, A. vaginae, Megasphaera, Leptotrichia, Sneathia, Prevotella*, *Lachnovaginosum* genomospecies (formerly *BVAB1*)*, BVAB2*, and *Mycoplasma*] were investigated. For these studies, the impact of exogenous sex steroids on the vaginal microbiota was reported as presence/absence, prevalence, and bacterial load (as mean target copies and log concentration) of key bacteria and change in these measurements stratified by sex steroid exposure over time. Studies that measured the vaginal microbiota with denaturing gradient gel electrophoresis (DGGE) and Sanger sequencing reported types and number of bacterial species present, but not relative abundance.

### Definitions of Outcomes

Findings were classified as positive, negative, or neutral and further qualified as significant or non-significant. Positive findings generally reflected an increase in *Lactobacillus* spp. among women with sex-steroid exposure. Despite controversy in the field surrounding the role of *L. iners* (Petrova et al., 2017), *L. iners* was grouped with other *Lactobacillus* spp. for the purposes of this review as it is still considered preferable over BV-associated bacteria. An exogenous sex steroid was designated to have a positive effect on the vaginal microbiota if they reported any of the following i) an increase in abundance and/or prevalence of *Lactobacillus* spp. (or decrease in BV-associated bacteria) following initiation of sex steroid use and/or relative to the control group (i.e. either baseline sample or no hormonal contraceptive-use group), ii) a change to a compositional state reflecting an optimal vaginal microbiota [defined as a vaginal microbiota dominated by lactobacilli such as CST-I, CST-II, CST-III (Ravel et al., 2011)], iii) maintenance of an optimal vaginal microbiota state over time, or iv) a decrease in bacterial diversity, specifically reflecting a low-diversity optimal vaginal microbiota state relative to the control group for that study. An exogenous sex steroid was designated as having a negative effect on the microbiota if it was associated with i) a loss of *L. crispatus* or any other *Lactobacillus* spp. relative to the control group, ii) an increase in prevalence and/or abundance of any BV-associated bacteria, iii) a change in compositional state reflecting a non-optimal state (e.g. one dominated/characterised by BV-associated bacteria such as CST-IV, CST-V), and iv) an increase in diversity associated with a higher diversity non-optimal state and/or instability of the vaginal microbiota, relative to the control group used in the study. When there was no significant change (or no difference between exposure and control group) in vaginal microbiota state, stability, or prevalence/abundance of bacterial species reported, an exogenous sex steroid was considered to have a neutral or inconclusive effect.

### Assessment of Bias and Quality

Two authors (L.K.R. and E.L.P.) assessed the risk of bias within studies and reported on i) representation of the general population, ii) intervention allocation, iii) sample size, iv) comparator group/s, v) stratification by intervention, vi) analysis adjusted for confounding variables and vii) methodology, as further defined in **Supplementary Table 2**. Studies were not excluded based on the risk of bias assessment.

## Results

### Study Selection

**Figure 1:**
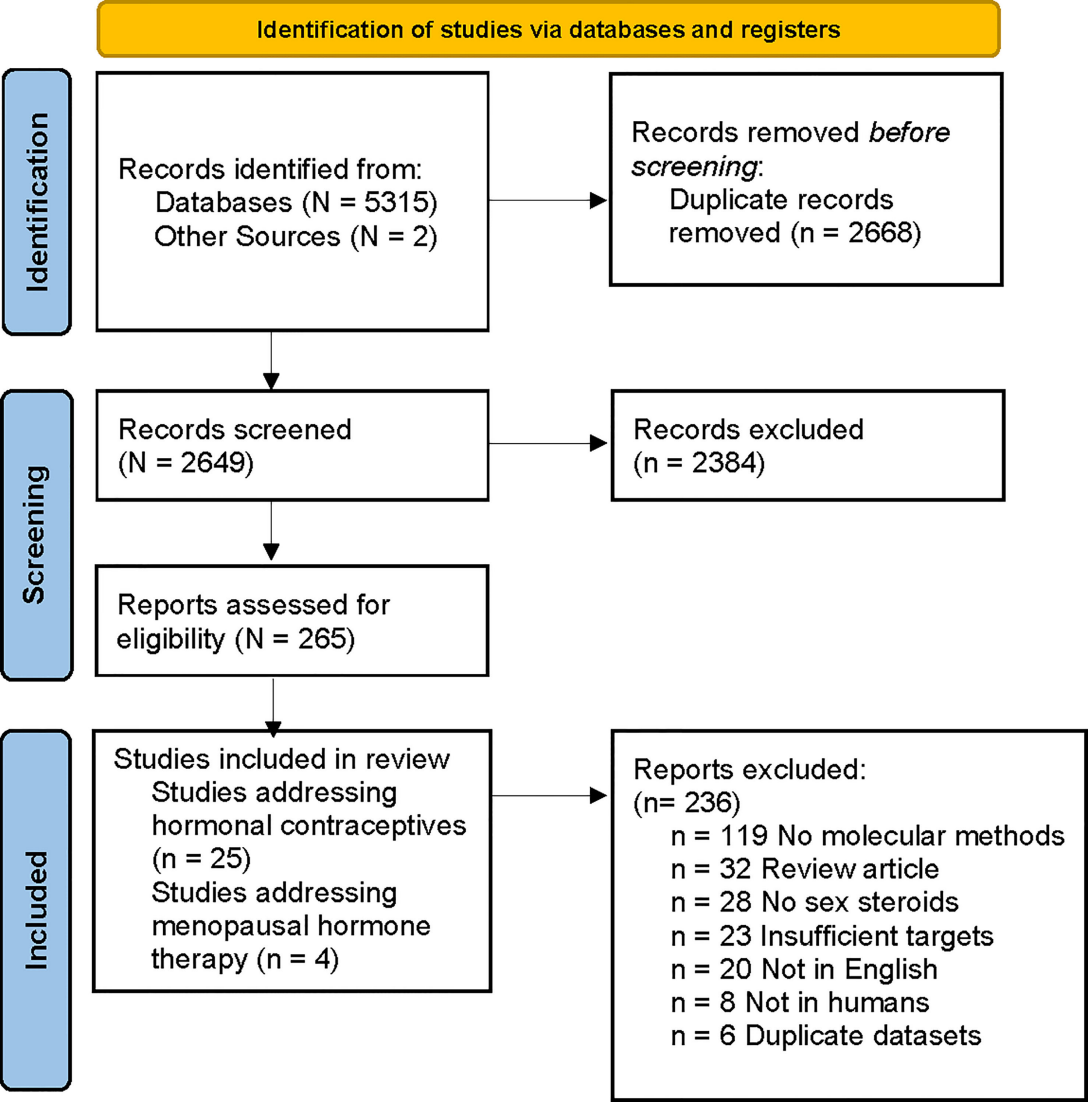
PRISMA flow diagram of literature search and article selection. Flow chart adapted from Page et al. (2021).

Literature searches identified 5315 records from Embase, Ovid Medline and PubMed; and two records were found through other sources. Duplicate references (n=2668) were removed, generating 2649 unique references (**Figure 1**). Of these, 2384 were excluded by title and abstract review. Of the remaining 265 articles, 236 were excluded during full-text screening. The reasons for excluding full text were: molecular methods not used (n=119), review article (n=32), article not available in English (n=20), insufficient bacterial targets (i.e. qPCR did not target BV-associated bacteria or *Lactobacillus* spp.) (n=23), no sex steroid outcomes reported (n=28), study not in humans (n=8) and datasets already represented in another publication (n=6). Twenty-nine eligible studies were identified and data extracted; with 25 studies reporting on the effect of hormonal contraception among reproductive-aged women (**Table 1**) and four on the effect of MHT among post-menopausal women (**Table 2**). Two studies reported on the same cohort; however, these were treated as separate studies as one reported on baseline data (Tuddenham et al., 2019a) and the second presented longitudinal data (Tuddenham et al., 2019b).

**Table 1:** Result summary of hormonal contraceptive studies.

First Author [ref]	Year	Location	Population and age if specified	Sample Size	Frequency of sampling	Molecular method used	Outcome Measure	Comparator Group	Hormone Source	Findings	Effect of exogenous sex steroids
Achilles et al. (2018)	2018	Harare, Zimbabwe	Women 18-35yo	266 women; 1047 samples	4x over 6 months	qPCR: 8 targets^a^	Prevalence and mean difference in quantity (expressed as gene copies/swab)	Baseline specimen^b^ (n=218)	Oestrogen-containing: ethinyl oestradiol injectable (n=40)	No changes in prevalence or log concentration after exposure to ethinyl oestradiol	Neutral effect of ethinyl oestradiol
									Progestin-only: DMPA (n=41), Net-En (n=44), LNG-implant (n=45), ENG-implant (n=48)	Initiation of DMPA associated with a decrease in log concentration of *L.iners*	Negative effect of DMPA
Balle et al. (2020)	2020	South Africa	Women willing to initiate or change HC, HIV negative 15-19yo	130 women and girls; 329 samples	3x over 32 weeks	MiSeq 16S V4 region	CST, Shannon diversity, differential abundance, community composition	Baseline specimen^b^ (n=130); alternate study arm	Oestrogen-containing: COCP (n=40), CVR (n=45)	COCP exposure associated with decreased abundance of *Prevotella*, *Mycoplasma*, *Sneathia* and *Parvimonas*; increased abundance of *L. iners*; lower Shannon diversity and likely to be CST-III compared to baseline.	Positive effect of COCP
										CVR-use no effect compared to baseline. CVR-use associated with high abundance of *Prevotella*, *Mycoplasma*, and *Parvimonas*, higher Shannon diversity and more likely to be CST-IV compared to COCP.	Neutral effect of CVR vs baseline
									Progestin-only: Net-En (n=45)	Net-En associated with higher abundance of *Prevotella*, *Sneathia*, and *Parvimonas*; more likely to be CST-IV compared to the COCP	Negative effect of Net-En
Bassis et al. (2015)	2015	Missouri, USA	Women at risk of unplanned pregnancy ≥18yo	76 women; 209 samples	3x over 12 months	MiSeq 16S V4 region	Distance metric (composition stability)	Baseline specimen^b^ (n=40) and Cu-IUD (n=36)	Progestin-only: LNG-IUS (n=40)	No changes to microbial composition over 12 months compared to baseline specimen and women using Cu-IUD	Neutral effect of LNG-IUS
Borgdorff et al. (2015)	2015	Kigali, Rwanda	Non-pregnant sex-workers	174 women; 196 samples	2x over 18 months	16S Microarray	Vaginal microbiota clusters and normalised relative abundance	Women using condoms (n=96)	Oestrogen-containing: OCP^c^ (n=49)	COCP use was not associated with changes in vaginal microbiota, non-significant decrease in *Sneathia* and *Prevotella* concentrations	Neutral effect of COCP
									Progestin-only: injectables^d^ (n=97)	Injectable use was not associated with any changes in vaginal microbiota	Neutral effect of DMPA
Borgdorff et al. (2017)	2017	Amsterdam, Netherlands	Non-pregnant women 18-34yo	610 women; 610 samples	N/A	MiSeq 16S V3-V4 regions	Richness (number of OTUs) and diversity (Shannon Diversity Index), vaginal microbiota groups (e.g. CSTs based on dominant taxa)	Women using condoms (n=439)	Oestrogen-containing: COCP & CVR (n=241^e^)	COCP and CVR use associated a non-significant decrease in *G.vaginalis*, but was overwhelmed by other factors in adjusted analyses	Non-significant positive effect of COCP
									Progestin-only: implant, hormonal patch, or injectable (n=241^e^)	Not investigated	N/A
Brooks et al. (2017)	2017	Virginia, USA	Non-pregnant women using condoms, COCP, DMPA or LNG-IUS 18-44yo	682 women; 682 samples	N/A	454 Pyro-sequencing 16S V1-V3 regions	Predominant taxon, abundance (mean %), alpha diversity (inverse Simpsons), Associations between species and contraceptives by LEfSE	Women using condoms (n=186)	Oestrogen-containing: COCP (n=206)	COCP use was associated with increased abundance of *Lactobacillus spp*, favouring an *L. crispatus* dominated CST and disfavouring a high-diversity CST, and decreased abundance of BV associated bacteria compared to condoms and progestin-only HCs	Positive effect of COCP
									Progestin-only: DMPA (n=94) and LNG-IUS (n=196)	DMPA and LNG-IUS were associated with a non-significant increase in BV associated bacteria compared to COCP	Neutral effect of DMPA and LNG-IUS
Crucitti et al. (2018)	2018	Rwanda	Non-pregnant, HIV negative sexually active women 18-35yo	120 women; 413 samples	5x over 3 months	qPCR: *7* targets^a^	Genome equivalents per ml of targets	Baseline specimen^b^ (n=120)	Oestrogen-containing: vaginal ring (n=120)	CVR use associated with *Lactobacillus* dominated vaginal microbiota cluster, increased prevalence of *L. crispatus, L. iners, L. jensenii, L. vaginalis* as well as decreased prevalence of *G.vaginalis* and *A.vaginae* compared to baseline	Positive effect of CVR
Dabee et al. (2019)	2019	Cape Town & Soweto	HIV negative women 16-22yo	59 women; 59 samples	N/A	16S MiSeq V4 region	*Lactobacillus spp* abundance vs other^f^ bacteria	Women not using contraceptives (n=5); condoms only (n=28)	Oestrogen-containing: COCP (n=4), CVR (n=1)	Not investigated	N/A
									Progestin-containing: DMPA (n=14) Nur-isterate (n=67) Net-En (n=37)	DMPA and Net-En use associated with loss of *Lactobacillus spp* compared to women not using progestin-containing contraceptives	Negative effect of DMPA and Net-En
Gautam et al. (2015)	2015	Kenya, Rwanda, Tanzania, South Africa	Non-pregnant, HIV negative women 16-35yo	230 women; 313 samples	enrolment & 81 follow-up samples	16S Microarray	Vaginal microbiota clusters and normalised abundance	Women using condoms, Cu-IUD or no contraceptives (n=103)	Oestrogen-containing: COCP (n=31)	Grouped HC-use^f^ not associated with any CST	Neutral effect of HCs^f^
									Progestin-only: DMPA (n=60)		
Jacobson et al. (2014)	2014	USA	Caucasian women 21-33yo	11 women; 406 samples	9x over 12 weeks	454 Pyro-sequencing 16S V1-V3 region	Relative abundance (proportion) and prevalence	Baseline specimen^b^ (n=11)	Progestin-only: LNG-IUS (n=11)	LNG-IUS was associated with a non-significant increase in *L.crispatus* compared to pre-IUS sample	Non-significant positive effect of LNG-IUS
Lennard et al. (2018)	2018	Cape Town & Johannesburg, South Africa	Black, non-pregnant, HIV negative women 16-22yo	185 women; 185 samples	N/A	16S MiSeq V4 region	Bray-Curtis diversity, relative abundance, Microbiota compositional and functional subtypes	Women using condoms (n=71)	Oestrogen-containing: OCP^c^ (n=10), VR (n=1)	Not investigated	N/A
									Progestin-only: DMPA (n=24), Net-En (n=70), Injectables (n=2)^d^, Implanon (n=7)	There was no significant difference in the proportion of DMPA and Net-En users assigned to each vaginal microbiota cluster compared to non HC users and other types of HC	Neutral effect of DMPA and Net-En
Marconi et al. (2020)	2020	Brazil	Non-pregnant, HIV-negative women 18-50yo	609 women; 609 samples	N/A	16S MiSeq V3-V4 region	CST	Women using condoms, women not using contraceptives (n=366)	Oestrogen-containing: OCP_1_ (n=192)	Grouped HC^f^ was associated with a decreased risk of CST-IV	Positive effect of HC^f^
									Progestin-only: Injectables^d^ (n=51)		
Onywera et al. (2019)	2019	Cape Town, South Africa	Heterosexual non-pregnant women with HIV or HIV/HPV 18-44yo	62 women; 62 samples	N/A	16S Ion Torrent V4 region	Diversity, community state types	Women using condoms, or not using contraception (n=37)	Oestrogen-containing: OCP^c^ (n=2)	OCP^c^ use was associated with non-significant lower diversity	Positive effect of OCP^c^
									Progestin-only: DMPA (n=18) and Net-En (n=5)	DMPA and Net-En use was associated with lower diversity and assignment to CST-I, CST-II and CST-III compared to non-users	Positive effect of DMPA and Net-En
Pyra et al. (2016)	2016	USA and Kenya	Non-pregnant HIV negative women 18-45yo	107 women; 107 samples	N/A	qPCR: 11 targets^a^	log load concentration	Women using condoms, Cu-IUD or no contracep-tion^g^	Oestrogen-containing: OCP^c,g^	Not investigated	N/A
									Progestin-only: Injectable^d, g^ and Implanon	Injectables were associated with lower prevalence of *L. iners* compared to women not using HC, but not compared to Cu-IUD users	Negative effect of injectables
Ratten et al. (2021)	2021	Australia	Women with BV 18-45yo	75 women; 430 samples	8x over 6 months	16S MiSeq V3-V4 region	Vaginal microbiota group type (lactobacillus vs non-lactobacillus dominated)	Women using condoms (n=39)	Oestrogen-containing: COCP (n=37)	COCP use did not significantly affect the vaginal microbiota compared to condom use	Non-significant positive effect of COCP
Scott et al. (2019)	2019	Los Angeles, California, USA	Pre-menopausal women	23 women; 276 samples	12 weeks	16S^h^ unknown region	Relative abundance, diversity, stability	Women not using HC^f^	COCP grouped HC^f,g^	HC^f^ use was associated with stability of the vaginal microbiota	Non-significant positive effect of HC^f^
Song et al. (2020)	2020	Wellesley, Massachusetts, USA	Female students 18-22yo	26 women; ~1100 samples^j^	2x a day for 10 weeks	16S MiSeq V3-V4 region	CST type, Lactobacillus abundance	Women using condoms, or not using contraception (n=16)	Oestrogen-containing: COCP and systemic^i^ contraceptives (n=9)	Oestrogen-containing contraceptives were associated with increased abundance of *Lactobacillus*	Non-significant positive effect of COCP and ‘C-Systemic’ contraceptives
									Progestin-only: local contraceptive^j^ (n=6)	Local release contraceptive use was associated with decreased abundance of *Lactobacillus* compared to oestrogen-containing contraceptives	Non-significant negative effect of local release progestin-only contraceptives
Thurman et al. (2019)	2019	USA & Dominican Republic	Women, BMI <30 kg/m2 18-45yo	51 women; 101 samples	2x over 7 visits	16S HiSeq V3-V4 region	CST, abundance and diversity	Baseline, pre tenofovir-LNG-CVR insertion sample	Progestin-only: tenofovir-LNG-CVR (n=20)	The proportion of women with an optimal vaginal microbiota (CST-I, CST-II and CST-V) increased following tenofovir-LNG-CVR insertion. There was a decrease in the proportion of women with a non-optimal vaginal microbiota (CST IV) following tenofovir-LNG-CVR insertion.	Positive effect of LNG-CVR
Tuddenham et al. (2019a)	2019	Baltimore, MD, USA	Reproductive aged women changing HC status	104 women; 104 samples	baseline only	16S HiSeq 2500 V3-V4 region	CST classification	Women not using HC (n=54)	Grouped HC^f^ (n=50)	HC^f^ use associated with assignment to CST-I, CST-II and CST-III	Positive effect of HC^f^
Tuddenham et al. (2019b)	2019	Baltimore, MD, USA	Reproductive aged women changing HC status	105 women; 4,185 samples	2x week for 2 weeks & 7x over 2 years	16S HiSeq 2500 V3-V4 region	CST classification	Women not using HC^f^ (n=32)	Grouped HC^f^ (n=73)	HC^f^ was associated with increased stability of the vaginal microbiota after >3 months of use and assignment to CST-I	Positive effect of HC^f^
van de Wijgert et al. (2020)	2020	Johannesburg, South Africa	Women with HIV and HPV	448 women; 847 samples	2x over 16 months	16S HiSeq 2500 V3-V4 region	Vaginal microbiota group classification relative abundance	Women using condoms/not having sex (n=333)	Oestrogen-containing: OCP^c^ (n=23)	There was no significant difference in the proportion of COCP users assigned to each vaginal microbiota compared to non-HC users	Neutral effect of COCP
									Progestin-only: DMPA (n=82)	There was no significant difference in the proportion of DMPA users assigned to each vaginal microbiota compared to non-HC users	Neutral effect of DMPA
Van Der Veer et al. (2019)	2019	Amsterdam, the Netherlands	Non-pregnant STI negative women 18-36yo	25 women; 1,061 samples	Daily for one menstrual cycle; every other day for two menstrual cycles	16S MiSeq V3-V4 region	Vaginal microbiota clusters	Women using condoms (n=10)	Oestrogen-containing: COCP (n=15)	COCP use was not associated with changes in the microbiota	Neutral effect of COCP
Wessels et al. (2019)	2019	Kenya	Sex workers with <5yrs sex work, pre-menopausal, STI and BV negative, non-pregnant or breastfeeding women ≥18yo	58 women; 58 samples	N/A	16S MiSeq V3 region	Alpha and Beta diversity, relative abundance	Women using condoms (n=22)	Oestrogen-containing: COCP (n=14)	COCP use was associated with an increase of >98% vaginal microbiota dominance by *Lactobacillus* compared to DMPA use	Positive effect of COCP
									Progestin-only: DMPA (n=22)	DMPA users had increased bacterial diversity by Shannon diversity index compared to COCP and condom use	Negative effect of DMPA
Whitney et al. (2020)	2020	Nairobi, Kenya	HIV negative breast-feeding women, 6–14 weeks postpartum seeking contraception	54 women; 98 samples	2x; baseline & month 3	qPCR: 7 targets^a^	Concentration of BVAB & pathogenic species	non-HC methods (condoms lactational amenorrhea, rhythm) (n=21)	Progestin-only: DMPA (n=33)	DMPA use was not associated with a significant difference prevalence or concentration of any taxa	Neutral effect of DMPA-IM
Yang et al. (2019)	2019	New Jersey, USA	Non-pregnant women with no HC exposure >20 months 18-35yo	25 women; 67 samples	3x over 3 months	16S MiSeq V3-V4 region	Alpha diversity. Differences in abundance and prevalence of BV associated bacteria vs *Lactobacillus spp* after starting DMPA.	Baseline specimen^b^ (n=25)	Progestin-only: DMPA (n=25)	DMPA was associated with increased diversity and lower *Lactobacillus* spp. abundance in black women and higher abundance of *Lactobacillus* spp. in white women compared to baseline specimen	Mixed effect of DMPA

NB. Contraceptives and acronyms are reported as per the original manuscript; where available, the variable region targeted in 16S rRNA sequencing is indicated (i.e. V3-V4, V1-V2); vaginal microbiota groups and clusters are as defined by authors.

AV, atrophic vaginitis; BV, bacterial vaginosis; COC, combined oral contraceptives; COCP, combined oestrogen-containing oral contraceptive pill; Cu-IUD, copper intrauterine device; CST, community state type; CVR, contraceptive vaginal ring; DGGE, denaturing gradient gel electrophoresis; DMPA, depo-medroxyprogesterone acetate; ENG, etonogestrel (implant); HC, hormonal contraceptive; LNG, levonorgestrel; LNG-IUS, levonorgestrel-releasing intrauterine system; MPA, medroxyprogesterone acetate; Net-En, norethisterone enanthatel; OCP, oral contraceptive pill; OTU, operation taxonomic units; qPCR, quantitative polymerase chain reaction; yo = years old.

a qPCR targets varied between studies and were reported as follows: Achilles (Achilles et al., 2018) L.crispatus, L.jensenii, L.gasseri/johnsonii, L.vaginalis, L.iners, G.vaginalis, A.vaginae and Megasphaera phylotype 1; Crucitti et al. (2018) L.iners, L.crispatus, L.jensenii, L.gasseri, L.vaginalis, A.vaginae & G.vaginalis; Pyra et al. (Pyra et al., 2016) Lachnovaginosum genomospecies [BVAB1], BVAB2, M.indolicus, A.vaginae, Megasphaera spp, Leptotrichia/Sneathia, G.vaginalis, L.crispatus, L.jensenii, and L.iners; and Whitney (Whitney et al., 2020) G.vaginalis, M.hominis, Sneathia spp, G.asaccharolytica, Eggerthella spp, Megasphaera spp and Parvimonas spp.;

b Baseline specimen is a specimen collected prior to HC-initiation;

c OCP not stratified by oestrogen-containing or progestin-only;

d Injectable, type not specified;

e number of samples (n) not stratified by HC-type within each sex steroid type;

f HC not stratified by oestrogen-containing or progestin-only;

g n not provided;

h Platform not specified;

i oestrogen and progestin combined, ‘systemic release’ contraceptives, not specified to maintain patient anonymity;

j progestin-only, ‘local release’ contraceptives, not specified to maintain patient anonymity.

**Table 2:** Summary of included menopausal hormone therapy studies.

Authors	Year	Location	Population	Sample Size	Sampling frequency	Method	Outcome Measure	Comparator Group	Hormone Source	Findings	Effect of exogenous sex steroids
Dahn et al. (2008)	2008	Ontario, Canada	Post-menopausal women using Permarin^®^ MHT for >2 months with age matched controls	20 women; 20 samples	Single specimen collected from each participant	DGEE and Microarray	Predominant lactobacillus and prevalence of species	Age matched women not on MHT (n=10)	conjugated oestrogen (n=10)	MHT was association with restoration of *Lactobacillus*	Positive effect of MHT
Devillard et al. (2004)	2004	Ontario, Canada	Post-menopausal women with no urogenital infections aged 41-66yo	19 women; 75 samples	4x over 90 days	DGGE and Sanger Sequencing	Prevalence (presence/absence) and no. of species detected (diversity)	Postmenopausal women not on MHT (n=20)^a^	conjugated oestrogen (n=19)	All women receiving MHT had *Lactobacillus* detected	Positive effect of MHT
Heinemann and Reid (2005)	2005	Canada	Postmenopausal women aged 41-82yo	60 women; 60 samples	4x over 3 months	DGGE and Sanger Sequencing	Prevalence of specific species	Women not using MHT (n=20)	conjugated oestrogen (n=40)	MHT increased the prevalence of *Lactobacillus*, stability of the vaginal microbiota and decrease of diversity	Positive effect of MHT
Shen et al. (2016)	2016	Shanghai, China	Post-menopausal women with genital symptoms, BMI between 18-35yo, non-smokers without *Trichomonas* or candida	59 women; 177 samples	3x over 4 weeks	16S MiSeq V1-V3	Phylotype abundance	Women not on MHT, without AV (n=29)	conjugated oestrogen (n=30)	MHT was associated with an increase of *Lactobacillus* abundance and decrease of diversity	Positive effect of MHT

NB. All studies investigated a conjugated-oestrogen formulation (topical Premarin^®^); where available, the variable region targeted in 16S rRNA sequencing is indicated (i.e. V1-V3).

AV, atrophic vaginitis; BMI, body mass index; DGGE, denaturing gradient gel electrophoresis; MHT, menopausal hormone therapy; yo = years old.

a The comparison group is post-menopausal women not on MHT who participated in another study.

### Studies Reporting on the Effect of Hormonal Contraceptives on the Vaginal Microbiota

#### General Characteristics

A summary of the populations included in the studies identified is provided in **Supplementary Table 3**. Of the 25 studies addressing the effect of hormonal contraception on the vaginal microbiota, 20 of these were in HIV negative, non-sex workers without BV, two were in women with HIV and human papillomavirus (HPV), two were in sex workers with no other risk factors specified, and one was among women with symptomatic BV. Nine studies were cross-sectional and 16 were longitudinal cohort studies; the median follow-up time was 31 weeks (range 7 days to 24 months). Nineteen of the studies investigated the vaginal microbiota using next-generation sequencing technology, four used qPCR and two used microarray. Twelve studies were conducted in sub-Saharan Africa, eight were conducted in North America, two in Europe/Central Asia, two were in Latin America/Caribbean and one was conducted in the East-Asian and Pacific region; there were no studies in South Asia or the Middle East/North Africa. These studies represent approximately 14,000 specimens from approximately 4,400 women; of which 1,048 women were exposed to combined oestrogen- and progestin-containing contraceptives (termed oestrogen-containing contraceptives) and 968 were exposed to progestin-only contraceptives (**Table 1**). The combined-oestrogen containing contraceptive-types reported on included i) oral contraceptive pill (OCP), ii) contraceptive vaginal ring (CVR) and iii) ethinyl oestradiol-containing injectable. The progestin-only contraceptive types included i) injectable depo-medroxyprogesterone acetate (DMPA), ii) injectable norethisterone enanthate (Net-En), and iii) levonorgestrel (LNG) contained with an intrauterine system (LNG-IUS) or CVR (LNG-CVR). Other progestin-only contraceptives, including subdermal contraceptive implants and patch-use, were identified, but there was no or limited data reported and so they were not included in the review. The remaining women were either not exposed to contraceptives/or used non-hormonal contraceptive methods (including condoms or the Cu-IUD). Most of the studies (15/25) identified investigated more than one type of hormonal contraception, and we have stratified the findings by sex steroid type where possible (**Table 1**).

#### The Effect of Oestrogen-Containing Hormonal Contraceptive Types on the Vaginal Microbiome

Fourteen studies investigated the effect of combined oestrogen- and progestin-containing contraceptives, which included the combined oestrogen-containing OCP, an oestrogen-containing CVR and the ethinyl oestradiol-containing injectable. When grouped together, 8/14 (57%) identified a positive effect of oestrogen-containing hormonal contraception on the vaginal microbiota, 5/14 (36%) found neutral or no effect, and one study found a negative effect (7%; **Table 3**).

**Table 3:** Summary of beneficial, neutral/inconclusive and detrimental effects of exogenous sex steroids on the vaginal microbiota.

	Overall effect on the vaginal microbiota (n = number of studies)		
Exogenous steroid delivery method	Positive	Neutral/Inconclusive	Negative
***Reproductive-aged women using HC***			
**All oestrogen-containing HC (n=14)**	8	5	1
** Oestrogen-containing OCP**^a^ (n=10)	6	4	0
**CVR** (n=3)	2	1	0
**Ethinyl oestradio injectable** (n=1)	0	1	0
**All progestin-only HC (n=20)**	4	8	8
**DMPA** (n=10)	1	6^b^	3
**Net-En** (n=4)	1	1	2
**LNG-IUS and LNG-CVR** ^c^ (n=4)	2	1	1
**Unspecified injectables** (n=1)	0	0	1
**Grouped ‘local release’ progestin-only contraceptives** (n=1)	0	0	1
**Grouped unspecified HC**^d^ (n=5)	4	1	0
***Post-menopausal women using MHT***			
**Conjugated-oestrogen formulation**	4	0	0

a A small proportion of oral contraceptive pill users may be using progestin-only pills, but this information was not available or authors suggested this was unlikely.

b Includes one paper with both positive and negative findings.

c The LNG-CVR investigated also contained tenofovir.

d includes studies that reported on hormonal contraceptives, but where the exogenous sex steroids are unspecified.

CVR, contraceptive vaginal ring; DMPA, Depo-medroxyprogesterone acetate; IUS, Intrauterine system; HC, hormonal contraception; LNG, levonorgestrel; MHT, menopausal hormone therapy; Net-En, norethisterone enanthatel; OCP, oral contraceptive pill.

##### Oestrogen-Containing Oral Contraceptive Pills

Ten studies investigated the effect of the combined oestrogen-containing OCP on the vaginal microbiome (Borgdorff et al., 2015; Borgdorff et al., 2017; Brooks et al., 2017; Onywera et al., 2019; Van Der Veer et al., 2019; Wessels et al., 2019; Balle et al., 2020; Song et al., 2020; van de Wijgert et al., 2020; Ratten et al., 2021); six reported a positive effect on the vaginal microbiota, and four reported neutral findings (**Tables 1** and **3**). The OCPs were specified as oestrogen-containing by the authors in all studies, except for two (Borgdorff et al., 2015; Onywera et al., 2019). However, for the purpose of this review, these two studies were assigned as studies of oestrogen-containing OCP, and this was confirmed by the authors to be the most common formulation. Four studies found OCP-use had a positive effect on *Lactobacillus* spp. (**Tables 1** and **3**); three studies reported that OCP-use was associated with a higher relative abundance of *Lactobacillus* spp. compared to women not using the OCP (Brooks et al., 2017; Song et al., 2020), or women’s baseline (i.e. pre-OCP initiation) specimens (Balle et al., 2020), and one reported an increase in the number of women with a vaginal microbiota dominated by *Lactobacillus* spp. (i.e. >98% relative abundance of *Lactobacillus* spp.) compared to women using condoms (Wessels et al., 2019). Balle et al. specifically identified that OCP-use increased the relative abundance of *L. iners* compared to participants’ baseline specimens (Balle et al., 2020). Onywera et al. (2019) and Balle et al. (2020) both reported that OCP-use decreased bacterial diversity (measured by the Shannon diversity index), compared to non-OCP users and baseline specimens, respectively. Balle et al. (2020) also found that OCP-use decreased the relative abundance of several BV-associated bacteria, specifically *Prevotella, Sneathia* and *Parvimonas* relative to baseline and Net-En users, while Borgdorff et al. (2017) found a non-significant decrease in the relative abundance of *G. vaginalis* in OCP-users *vs* non-OCP users. Four studies found no effect of the OCP on the vaginal microbiota compared to women using condoms (Borgdorff et al., 2015; Van Der Veer et al., 2019; van de Wijgert et al., 2020; Ratten et al., 2021).

##### Oestrogen-Containing Contraceptive Vaginal Ring

Three studies investigated the effect of the oestrogen-containing CVR (Borgdorff et al., 2017; Crucitti et al., 2018; Balle et al., 2020). Compared to baseline specimens, Borgdorff et al. (2017) found that CVR-use was associated with a non-significant decrease in the relative abundance of *G. vaginalis* and Crucitti et al. (2018) found CVR-use was associated with a significant decrease in the prevalence *G.vaginalis* and *A.vaginae* (**Table 1**). Crucitti et al. (2018) also found that CVR-use was associated with an increase in the prevalence and load of *Lactobacillus* spp. in the vaginal microbiota, specifically *L. crispatus, L. iners, L. jensenii* and *L. vaginalis*. Conversely, Balle et al. (2020) found that oestrogen-containing CVR use had no impact on bacterial diversity compared to baseline. However, compared to OCP users, CVR-use was associated with a higher relative abundance of *Prevotella, Mycoplasma*, and *Parvimonas*, higher bacterial diversity and increased likelihood of having a vaginal microbiota composition designated to CST-IV (Balle et al., 2020).

##### Ethinyl Oestradiol Containing Injectable

Only one study investigated the effect of the ethinyl oestradiol-containing injectable among 40 women in Zimbabwe (Achilles et al., 2018). There were no significant changes to the composition of the vaginal microbiota following ethinyl oestrodial-containing injectable use relative to baseline specimens.

#### The Effect of Progestin-Only Contraceptives on the Vaginal Microbiotas

Twenty studies investigated the effect of progestin-only contraceptives, representing DMPA, Net-En and the levonorgestrel CVR and IUS. When looked at together, four studies identified a positive effect of progestin-only contraceptives on the vaginal microbiota, nine described a negative effect on the microbiota, one study reported different effects across patient sub-populations, and seven reported neutral or inconclusive effects (**Tables 1** and **3**).

##### Depot-Medroxyprogesterone Acetate Injectable

Of the ten studies that investigated DMPA (Borgdorff et al., 2015; Brooks et al., 2017; Achilles et al., 2018; Lennard et al., 2018; Dabee et al., 2019; Onywera et al., 2019; Wessels et al., 2019; Yang et al., 2019; van de Wijgert et al., 2020; Whitney et al., 2020), three reported a negative effect of DMPA on the vaginal microbiota, one reported a positive effect, five reported neutral findings and one reported mixed findings. DMPA-use was associated with a decrease in the relative abundance of *Lactobacillus* spp. in two studies (additional data provided by K. Lennard) (Dabee et al., 2019; Yang et al., 2019). In the study by Yang et al, DMPA-use had a different effect in the two study populations; it was associated with a decreased abundance of *Lactobacillus* spp. among African American/black women and an increase in relative abundance of *Lactobacillus* spp. among Caucasian/white women (Yang et al., 2019). Achilles et al. (2018) also reported a loss of *Lactobacillus* spp., specifically a decrease in log concentration of *L. iners* over six months of DMPA-use. Brooks et al. (2017) described a non-significant increase in the relative abundance of BV-associated bacteria associated with DMPA-use, which was classified as an inconclusive finding. Wessels et al. (2019) found that DMPA users had an increase in bacterial diversity (measured by Shannon diversity index). Conversely, Onywera et al. (2019) found that DMPA-use was associated with lower bacterial diversity (measured by Shannon diversity index) and a vaginal microbiota assigned to *Lactobacillus* spp. dominated CSTs (CST-I, CST-II and CST-III) relative to women using condoms or no contraceptives. One study found that DMPA had no effect on the prevalence and concentrations of BV-associated bacteria *Sneathia, M. hominis* and *Parvimonas* species type 1 *vs* women who did not use contraception/used non-hormonal contraceptives (Whitney et al., 2020). However, this study did not specifically look at the effect of DMPA on lactobacilli. Three studies found that there was no significant difference in the proportion of DMPA-users assigned to specific vaginal microbiota groups associated with optimal or non-optimal compositions compared to non-hormonal contraceptive users and/or users of other hormonal contraceptive types (additional data provided by [Lennard et al. (2018), van de Wijgert et al. (2020)] (Borgdorff et al., 2015; Lennard et al., 2018; van de Wijgert et al., 2020). In addition, Pyra et al. (2016) investigated the effect of injectable contraceptives but did not specify the type used. They defined injectable contraceptives as having a negative effect, as their use was associated with a lower prevalence of *L. iners*. Overall, DMPA had mixed effects with nearly equal numbers of studies reporting positive or neutral effects and negative effects on the vaginal microbiota.

##### Norethisterone Enanthate Injectable

Four studies investigated the effect of Net-En on the vaginal microbiota (Lennard et al., 2018; Dabee et al., 2019; Onywera et al., 2019; Balle et al., 2020), two of which had negative findings, one had positive findings and one had neutral findings. Balle et al. (2020) compared the effects of Net-En with participants’ baseline samples on the vaginal microbiota and found no effect of Net-En initiation. However, Net-En users were also more likely to be assigned to CST-IV than participants using the combined oestrogen-containing OCP (Balle et al., 2020). Dabee et al. (2019) found use of Net-En was associated with lower relative abundance of *Lactobacillus* spp. compared to women not using any hormonal contraception. In contrast, Onywera et al. (2019) found that Net-En use was associated with lower bacterial diversity (measured by Shannon diversity index) and assignment to *Lactobacillus* dominated CSTs compared to women not using hormonal contraception, however this represented a small proportion of Net-En users captured in this review (n=5). Lennard et al. (2018), had the most Net-En users (n=70) in their study and found that Net-En use was not associated with any specific vaginal microbiota composition when compared to women not using hormonal contraception, or women using other types of hormonal contraception (additional data provided by K. Lennard).

##### Levonorgestrel-Containing Contraceptive Rings and Intra-Uterine Systems

Four studies investigated the effect of levonorgestrel-containing systems on the vaginal microbiota (Jacobson et al., 2014; Bassis et al., 2015; Brooks et al., 2017; Thurman et al., 2019), two of which had positive findings, one had negative findings and the other had neutral findings. Thurman et al. (2019) found that the proportion of women with an optimal vaginal microbiota (CST I, II and V) increased following tenofovir-LNG-CVR insertion. There was a decrease in the proportion of women with a non-optimal vaginal microbiota (CST IV) following tenofovir-LNG-CVR insertion (Thurman et al., 2019). Three studies investigated the LNG-IUS with discordant findings; Jacobson et al. (2014) found that following insertion of LNG-IUS, the relative abundance of *L. crispatus* increased. Conversely, Brooks et al. (2017) compared LNG-IUS use to OCP-use and found a non-significant change in the relative abundance of BV-associated bacteria, while Bassis et al. (2015) found no difference in the vaginal microbiota composition between women using LNG-IUS and those using a Cu-IUD.

Song et al. (2020) grouped contraceptives as either oestrogen-containing or combined progestin-only “local release” contraceptives due to the small sample size (n=7, n=4 respectively). Compared to women not using hormonal contraception, oestrogen-containing contraceptives were associated with a non-significant increase in *Lactobacillus* spp. and use of progestin-only contraceptives was associated with a non-significant decrease in on the abundance of *Lactobacillus* spp (Song et al., 2020).

##### Studies That Grouped All Hormonal Contraceptives Together and Did Not Specify Type

Five studies did not stratify findings by hormonal contraception type (**Tables 1** and **3**; four reported a positive effect of grouped hormonal contraception-use on the vaginal microbiota and one reported a neutral effect. Scott et al. (2019) and Tuddenham et al. (2019b) found that grouped hormonal contraception-use was associated with stability of the vaginal microbiota. Both the cross-sectional and longitudinal studies by Tuddenham et al. (Tuddenham et al., 2019a; Tuddenham et al., 2019b) found that hormonal contraception-use was associated with a vaginal microbiota defined by optimal CSTs, and Marconi et al. (2020) found that hormonal contraception-use was associated with a decreased risk of having CST-IV. Gautam et al. (2015) reported that hormonal contraception-use did not change the vaginal microbiota.

### Studies Reporting on the Effect of Menopausal Hormone Therapy on the Vaginal Microbiota

#### General Characteristics

Four studies that investigated the effect of MHT on the vaginal microbiota were identified (**Tables 2** and **3**) (Devillard et al., 2004; Heinemann and Reid, 2005; Dahn et al., 2008; Shen et al., 2016). These studies represent 332 samples from 158 women, and all four assessed the effect of a topical conjugated-oestrogen containing formulation (topical Premarin^®^). All studies were in post-menopausal women, with three studies performed in Canada (Devillard et al., 2004; Heinemann and Reid, 2005; Dahn et al., 2008) and one in China (Shen et al., 2016) (**Supplementary Table 3**). One study was cross-sectional (Dahn et al., 2008) and the other three were longitudinal (Devillard et al., 2004; Heinemann and Reid, 2005; Shen et al., 2016); the median follow up time was 10 weeks (range 4-13 weeks). Three studies used DGGE based methods (Devillard et al., 2004; Heinemann and Reid, 2005; Dahn et al., 2008); two were in combination with Sanger sequencing (Devillard et al., 2004; Heinemann and Reid, 2005) one used microarray. Shen et al. (2016) was the only study to use next-generation sequencing (**Supplementary Table 3**).

#### The Effect of Menopausal Hormone Therapy on the Vaginal Microbiota

All four studies found that use of topical conjugated-oestrogens increased the prevalence of *Lactobacillus* spp. compared to women not using menopausal hormone therapy (Devillard et al., 2004; Heinemann and Reid, 2005; Dahn et al., 2008; Shen et al., 2016) (**Tables 2** and **3**). Shen et al. (2016) and Heinemann and Reid (2005) also found that conjugated-oestrogen use decreased the bacterial diversity of the vaginal microbiota and in addition, Heinemann and Reid (2005) reported that its use increased the stability of the vaginal microbiota over time.

#### Assessment of Study Bias

We assessed each of the studies for Selection Bias, Sample size and Measurement Bias across 6 domains, with a summary score for risk of bias generated (**Supplementary Table 2**). Studies with the lowest summary score were considered to have the lowest risk of study bias, however none of the included studies had a low risk of bias across all criteria assessed (**Table 4**).

**Table 4:** Risk of Bias Summary Table.

First author [ref]	Year	Location/Country	Selection Bias		Sample Size	Measurement Bias			*Summary of the overall risk of study bias*
			Representative of the general population?	Randomly allocated?	Adequate sample size?	Comparator Group?	Stratified by hormone?	Adjusted?	
*Studies of the effect of Hormonal Contraceptives*									
Achilles et al. (2018)	2018	Zimbabwe	0	2	0	1	0	1	4
Balle et al. (2020)	2020	South Africa	1	0	0	1	0	0	2
Bassis et al. (2015)	2015	USA	1	2	0	1	0	1	5
Borgdorff et al. (2015)	2015	Rwanda	1	2	0	0	0	0	3
Borgdorff et al. (2017)	2017	Netherlands	0	2	0	0	0	0	2
Brooks et al. (2017)	2017	selected from VaHMP	0	2	0	0	0	0	2
Crucitti et al. (2018)	2018	Rwanda	0	2	0	1	0	1	2
Dabee et al. (2019)	2019	Cape Town & Soweto	0	2	1	0	0	0	3
Gautam et al. (2015)	2015	Africa	1	2	0	0	1	1	5
Jacobson et al. (2014)	2014	USA	0	2	1	1	0	0	4
Lennard et al. (2018)	2018	South Africa	1	2	0	0	0	0	3
Marconi et al. (2020)	2020	Brazil (multiple sites)	0	2	0	0	1	0	3
Onywera et al. (2019)	2019	Cape Town, South Africa	1	2	1	0	1	1	6
Pyra et al. (2016)	2016	Multiple countries	0	2	0	0	0	0	2
Ratten et al. (2021)	2020	Australia	1	0	1	0	0	0	2
Scott et al. (2019)	2019	USA	?*	2	1	0	1	0	4
Song et al. (2020)	2020	Wellesley, Massachusetts, USA	1	2	1	0	0	1	5
Thurman et al. (2019)	2019	USA and Dominican Republic	0	0	1	0	0	0	1
Tuddenham et al. (2019a)	2019	Baltimore, MD, USA	0	2	0	0	1	1	4
Tuddenham et al. (2019b)	2019	Baltimore, MD, USA	0	2	0	1	1	1	5
van de Wijgert et al. (2020)	2019	Johannesburg, South Africa	1	2	0	0	0	0	3
Van Der Veer et al. (2019)	2019	Amsterdam, the Netherlands	0	2	1	0	0	0	3
Wessels et al. (2019)	2019	Kenya	1	2	1	0	0	1	5
Whitney et al. (2020)	2020	Nairobi, Kenya	1	2	1	0	0	0	4
Yang et al. (2019)	2019	New Jersey, USA	0	1	1	1	0	0	3
*Studies of the effect of Menopausal Hormone Therapy*									
Dahn et al. (2008)	2008	Canada	?*	2	1	0	0	1	4
Devillard et al. (2004)	2004	Multiple countries	0	2	1	2	0	1	6
Heinemann and Reid (2005)	2005	Canada	1	2	1	0	0	1	5
Shen et al. (2016)	2016	China	1	2	1	2	0	1	7

*Patient population characteristics not described.

##### Studies Reporting on the Effect of Hormonal Contraceptives on the Vaginal Microbiota Among Reproductive-Aged Women

When looking at selection bias across the 25 studies that investigated hormonal contraception, the study population was not described for only one study (Scott et al., 2019). One study was conducted on samples from a randomised control trial (Ratten et al., 2021) and in one, study participants were assigned treatment sequentially (Yang et al., 2019). For the remaining 22 studies, the participants self-selected their intervention. Eleven studies included a sample size of <100 participants total, which we assessed as high risk of bias (Jacobson et al., 2014; Dabee et al., 2019; Onywera et al., 2019; Scott et al., 2019; Van Der Veer et al., 2019; Yang et al., 2019; Song et al., 2020; Ratten et al., 2021). Eighteen studies used age-matched women not using hormonal contraceptives as the comparator group (e.g. no contraceptives or condom-use) (low risk), and seven used the baseline specimen prior to contraception initiation as the comparator group (low/medium risk) (Jacobson et al., 2014; Bassis et al., 2015; Achilles et al., 2018; Crucitti et al., 2018; Tuddenham et al., 2019b; Yang et al., 2019; Balle et al., 2020). Five studies did not stratify hormonal contraceptive exposure by oestrogen-containing or progestin-only (high risk) (Gautam et al., 2015; Scott et al., 2019; Tuddenham et al., 2019a; Tuddenham et al., 2019b; Marconi et al., 2020). Nine studies did not adjust analyses for confounding variables such as sex and douching practices (high risk) (Bassis et al., 2015; Gautam et al., 2015; Achilles et al., 2018; Crucitti et al., 2018; Tuddenham et al., 2019a; Tuddenham et al., 2019b; Onywera et al., 2019; Wessels et al., 2019; Song et al., 2020).

##### Studies Reporting on the Effect of Menopausal Hormone Therapy on the Vaginal Microbiota

Of the four MHT studies, one study did not define the study population (unknown risk) (Dahn et al., 2008), one was conducted in healthy women (classified as low risk or bias) (Devillard et al., 2004), and two were conducted in women with urogenital symptoms or infection (medium risk) (Heinemann and Reid, 2005; Shen et al., 2016). Women self-selected their therapy in all four studies, and all studies had fewer than 100 women and did not demonstrate sample size calculations for the effect of MHT use on the vaginal microbiota (high risk). Two studies had age-matched MHT-free women as comparators (low risk) (Dahn et al., 2008; Shen et al., 2016). In one study the comparator group was women who were not using MHT who had participated in another study (high risk) (Devillard et al., 2004). The fourth study compared findings to a group of women not using MHT, but these women had different symptoms to the MHT-exposed group (high risk) (Shen et al., 2016). All studies were stratified by hormone source (low risk), but no study adjusted for confounding variables (high risk).

## Discussion

Exogenous sex steroids contained within hormonal contraceptives and menopausal hormone therapy have been used principally for family planning and management of menopausal symptoms, without consideration for potential effects on the vaginal microbiota. This is because their use in healthcare predates our current understanding of the importance of the vaginal microbiota in human health, largely due to the fact that they were adopted before the technology existed to assess the vaginal microbiota. Prior systematic reviews and a meta-analysis of observational data found that oestrogen-containing contraceptives in particular have a beneficial effect on the vaginal microbiota, as measured by non-molecular methods (van de Wijgert et al., 2013; Vodstrcil et al., 2013). In this systematic review, we aimed to determine the effects of exogenous sex steroid use on the vaginal microbiota, as measured using modern, molecular-based methods such as high-throughput sequencing. We found that oestrogen-containing contraceptives, particularly the combined oestrogen and progestin-containing OCP, had a positive effect on the composition of the vaginal microbiota. Among post-menopausal women using MHT, exogenous oestrogen also appeared to positively influence the vaginal microbiota. However, the significance of an optimal vaginal microbiota as defined in reproductive-aged women is not as well understood in the post-menopausal population. In contrast, contraceptives containing progestin alone had mixed effects on the vaginal microbiota of reproductive-aged women. Overall, our systematic review shows that oestrogen may play a role in supporting an optimal vaginal microbiota in both reproductive aged and peri/post-menopausal women. However, further well-powered studies with appropriate control groups are required to explore the specific effects of different oestrogen-containing and progestin-only contraceptives.

### The Impact of Exogenous Oestrogen on the Vaginal Microbiota

We found that exogenous oestrogen, which predominantly reflected the use of the combined oestrogen and progestin-containing OCP, had a positive impact on the vaginal microbiota composition. Specifically, there was an increase in the prevalence and abundance of *Lactobacillus* spp. observed following oestrogen-exposure. *Lactobacillus* spp., particularly *L. crispatus*, characterise a vaginal microbiota associated with optimal reproductive and sexual health outcomes (Petrova et al., 2015; Anahtar et al., 2018). The positive effect of lactobacilli is proposed to be due to the production of lactic acid, which lowers the vaginal pH in addition to conferring antimicrobial and immunomodulatory benefits, and inhibiting the growth of anaerobic bacteria (Aldunate et al., 2015; Hearps et al., 2017; Tachedjian et al., 2017; Tyssen et al., 2018; Delgado-Diaz et al., 2020; Plummer et al., 2021). Different lactobacilli can produce two different lactic acid isomers, the L-isomer and D-isomer. The D-isomer is hypothesised to offer more protection against some bacterial upper genital tract infections such as chlamydia, however the L-isomer is more potent in inactivating HIV compared to the D-isomer at threshold concentrations *in vitro* (O’Hanlon et al., 2011; Aldunate et al., 2013; O’Hanlon et al., 2013; Witkin et al., 2013; Witkin, 2018; Edwards et al., 2019). In reproductive-aged women, endogenous oestrogen stimulates glycogen production by epithelial cells, which is then metabolised by *Lactobacillus* spp. as an energy source resulting in production of lactic acid (Boskey et al., 2001; O’Hanlon et al., 2011; O’Hanlon et al., 2013; van der Veer et al., 2019; Clabaut et al., 2021). Among women taking exogenous oestrogen, the amount of free glycogen available may increase or be more consistent throughout the menstrual cycle, and in turn indirectly increase lactic acid production (Mirmonsef and Spear, 2014; Nunn and Forney, 2016). Exogenous oestrogen could therefore have a therapeutic role among reproductive-aged women with a paucity of *Lactobacillus* spp. in their vaginal microbiota, such as in women with BV and vaginitis. In the one pilot randomised controlled trial to randomise women with BV receiving antibiotic treatment to adjunctive combined oestrogen-containing OCP-use or no OCP-use, there was no significant effect of sex steroid-exposure on BV recurrence rates (Vodstrcil et al., 2019). However, the findings of this study were impacted by the small sample size and attrition, and larger studies may be required to help us determine whether the use of oestrogen-containing contraceptives adjunctively or alone may positively influence the vaginal microbiome of women with BV.

The oestrogen-containing CVR had mixed impacts on the vaginal microbiota. Oestrogen-containing CVR-use was associated with an increase in several *Lactobacillus* spp. including *L. crispatus, L. iners, L. vaginalis* (Crucitti et al., 2018) and decrease in BV-associated bacteria such as *G. vaginalis* (Borgdorff et al., 2017; Crucitti et al., 2018). However, oestrogen-containing CVR-use was also shown to have no effect (Balle et al., 2020). Similarly, among women using progestin-only CVRs and intrauterine systems, the composition of the vaginal microbiota varied (Jacobson et al., 2014; Bassis et al., 2015; Brooks et al., 2017; Thurman et al., 2019). The high variability between these studies may be because of the limited number of studies, the different control groups used and/or the timing of the post-insertion specimens relative to when the contraceptive system was inserted. More studies are needed to understand the impact of CVR-use and IUS-use on vaginal microbiota composition.

Topical conjugated oestrogens in post-menopausal women was also associated with an increase in the abundance and prevalence of *Lactobacillus* spp. While there is abundant evidence to support the use of oestrogen to relieve post-menopausal symptoms such as vaginal dryness and discomfort (Hickey et al., 2016), we identified only four studies investigating menopausal hormone therapy on the vaginal microbiota. As there is limited information about the role of the vaginal microbiota in post-menopausal women, we do not know if there are benefits associated with re-establishing a *Lactobacillus* dominant vaginal microbiota in post-menopausal women.

### The Effect of Progestin on the Vaginal Microbiota in Reproductive-Aged Women

There is global interest in how progestin-only hormonal contraception, especially DMPA, might impact the vaginal microbiota and may increase the risk of STI and/or HIV acquisition. Our systematic review found that the effect of progestin-only contraceptives on the vaginal microbiota was mixed. Of note, half (n=11) of these papers examined DMPA-use. In contrast to oestrogen-containing contraceptives, progestin-only contraceptives had an inconsistent effect on the abundance and prevalence of *Lactobacillus* spp. as well as other metrics such as bacterial diversity and prevalence/abundance of BV-associated bacteria. The inconsistent findings of the effect of progestin-only contraceptives on the vaginal microbiota within and/or between sub-populations may be explained by host genetics and gene polymorphisms (Dabee et al., 2021), however further research is needed. The effect of DMPA on the vaginal microbiota is of particular interest due to its high rate of use in sub-Saharan Africa, and concerns it may enhance HIV transmission/acquisition (Galvin and Cohen, 2004; Curtis et al., 2020; Smith et al., 2020). This was reflected in our study screening process, which identified most progestin-only studies investigated the impact of DMPA on the vaginal microbiota. These studies were predominantly of women in sub-Saharan Africa, where DMPA usage coincides with high rates of HIV. Other possible detrimental effects following initiation of DMPA include epithelium thinning in the vagina, tissue inflammation and altered cell-mediated immune responses. In fact all of these effects increase a woman’s susceptibility to BV, HIV and other STIs (van de Wijgert et al., 2013; Murphy et al., 2014). Using previously published microbiome data from the CAPRISA-004 trial (Klatt et al., 2017), women with a *Lactobacillus-*dominant vaginal microbiota (primarily reflecting *L. iners*) who were using DMPA had a 3-fold increased risk of HIV acquisition (relative to women using Net-En or OCP) (Noël-Romas et al., 2020). In addition, higher serum-MPA concentrations were associated with evidence of inflammation in the vaginal mucosal fluid of *Lactobacillus-*dominated women (Noël-Romas et al., 2020). Interestingly, these effects of DMPA and serum-MPA were not observed in non-*Lactobacillus-*dominant women. The authors concluded that there is an interaction between the microbiome, hormonal contraceptives, and HIV susceptibility, demonstrating the importance of the both the vaginal microbiota and hormonal contraceptives in assessing HIV risk. Indeed, specific BV-associated bacteria themselves have been shown to enhance inflammatory pathways, which further complicates our understanding of these relationships (Dabee et al., 2021). The Evidence for Contraceptive Options in HIV Outcomes (ECHO) trial went on to assess if the risk of HIV differed with the use of three contraceptive methods – DMPA, LNG implant and Cu-IUD - but found no substantial difference in risk of HIV acquisition by contraceptive method used (Ahmed et al., 2019). The results from subsequent molecular analyses from the ECHO trial are pending and will be of great interest due to the concern regarding the possible impact of contraceptive practices on the risk of HIV acquisition and transmission. Clearly the relationship between progestin-only contraceptive use and the vaginal microbiota are more complex than for oestrogen-containing contraceptives, and require further investigation in the context of different populations and settings, as observed in the study by Yang et al. (2020).

### Strengths and Limitations

Our systematic review identified a significant number of studies that reported on hormonal contraception-use on the vaginal microbiota in reproductive aged women (n=25). We restricted our inclusion criteria to studies that measured the vaginal microbiota by molecular methods to reduce the significant bias which can be introduced by culture-dependant methods and microscopy. These methods only identify organisms that are able to be cultivated or are limited by taxonomic resolution. Despite the differences in methods used, next-generation sequencing was the most common measurement recorded in this review (n=20). However, all molecular methods have different biases, from DNA extraction, to data analysis, which should be considered. For example, different approaches to the steps used in 16S rRNA gene sequencing pipelines (i.e. DNA extraction method, primer selection, variable region selection, 16S rRNA gene reference database etc) can result in different findings when applied to the same samples (Pollock et al., 2018; Balkus et al., 2019). Additionally, microarray and qPCR results are dependent on the panel of targets tested. These factors further limited our ability to compare findings across studies. This study had a number of additional limitations. In the reproductive-aged cohort, most studies were conducted in sub-Saharan Africa (n=12) and North America (n=8) and therefore the effects of hormonal contraceptives may not be generalizable to other populations. This was even more apparent in the post-menopausal cohort where three of the four studies included were conducted in Canada, with the fourth from China. The small number of studied population groups, and the diverse findings across studies limited further comparison of our findings. Our analysis of bias also identified several other limitations specific to each of the included studies (**Table 4**). There was a high degree of variation in how the studies were conducted. For example, the contraceptive types assessed, how long contraceptives were used for, if there was a wash-out period from previous exogenous steroid exposure, the sample size, and how the data were analysed. The comparator groups also varied and included the participants’ baseline specimens or specimens from patients using non-hormonal contraceptives such as condoms or a Cu-IUD. The use of a Cu-IUD as the control group may have overestimated the benefit of the hormonal contraceptive assessed, as Cu-IUD use itself has been associated with dysbiosis (Erol et al., 2014; Achilles et al., 2018). Notably, many studies had small sample sizes, and, in most cases, patients self-selected their exposure and self-reported adherence to use. Furthermore, the effects of exogenous sex steroid exposure were not adjusted for behavioural practices. Despite contacting authors to retrieve more information, several studies were unable to specify which hormonal contraception-types were included in their hormonal contraception-use group/s, which prevented our ability to include the studies in analyses stratified by sex steroid-type. Regardless, of the five studies which included unspecified hormonal contraceptive-types, four found a positive influence of their use on the vaginal microbiota. Finally, there are newer formulations of hormonal contraceptives and menopausal hormone therapy that were not captured in this review.

### Conclusions

Access to contraceptives is an invaluable part of modern women’s healthcare, but until recently there was little consideration as to how this might affect the vaginal microbiota. With increasing use of hormonal contraceptives and menopausal hormone therapy globally, and improved understanding of how the microbiota may contribute to negative reproductive and gynaecological outcomes, it is important we understand the immediate and long-term impact of specific exogenous sex steroid use on the vaginal microbiota. Advances in high throughput DNA sequencing has made molecular analyses of the vaginal microbiota accessible and allowed us to gain more insight into the effects exogenous sex steroids may have on specific organisms, as well as its overall composition.

Our findings suggest that oestrogen-containing contraceptives and MHT may promote an optimal vaginal microbiota in some populations, which could have clinical applications, such as adjunctive therapy to improve management of BV and vaginitis (atrophic). While research is needed to support the use of exogenous-oestrogen as an adjunctive therapy, our data suggests it does not impact the composition of the vaginal microbiota in a detrimental way. The impact of progestin-only hormonal contraceptives was less consistent as there was equal evidence that they have either a negative or neutral impact on the vaginal microbiota. Clearly, more data is needed in order to confirm that DMPA and other progestin-only contraceptives are not contributing to adverse reproductive and sexual health outcomes. The molecular results from the ECHO trial are greatly anticipated and may provide some answers. Additional prospective studies from a wider range of populations that identify the underlying mechanisms by which progestin-only contraceptives alter the vaginal microbiota are also needed.

In summary, this review highlights the complex nature of the relationship between progestin-only contraceptives and the vaginal microbiome, and confirms the potential benefits of exogenous oestrogen in conferring a vaginal microbiota associated with optimal health outcomes for women.

## Data Availability Statement

The original contributions presented in the study are included in the article/**Supplementary Material**. Further inquiries can be directed to the corresponding authors.

## Author Contributions

LR, LV, and CB conceived and designed the study. LR and EP conducted the formal analysis, extracted all data, and interpreted the data, with support from LV and CB. GM provided advice around molecular technologies used. LM, GT, and DB provided additional data interpretation. CB, LV, GM, CF, and SG provided supervision. LR drafted the initial manuscript with supervision from LV, and EP, CB, CF, GM, SG, DB, GT, and LM. All authors contributed to the article and approved the submitted version.

## Funding

EP is supported by an Australian Government Research Training Program Scholarship. CF, CB, and SG are supported by Australian NHMRC Leadership Investigator Grants (GNT1172900, GNT1173361, and APP1197951, respectively). GT was supported by NHMRC Senior Research Fellowship (GNT1117748) and NHMRC Project Grant (GNT1164982).

## Conflict of Interest

GT is a co-inventor on patent application AU201501042 and United States Patent No. US 9,801,839 B2 claiming the anti-inflammatory effects of lactic acid. DB has attended advisory meetings and provided educational updates for clinicians for MSD and Bayer Healthcare as part of her role as Medical Director at FPNSW but has never received personal remuneration for these services.

The remaining authors declare that the research was conducted in the absence of any commercial or financial relationships that could be construed as a potential conflict of interest.

## Publisher’s Note

All claims expressed in this article are solely those of the authors and do not necessarily represent those of their affiliated organizations, or those of the publisher, the editors and the reviewers. Any product that may be evaluated in this article, or claim that may be made by its manufacturer, is not guaranteed or endorsed by the publisher.
