# Supplementary material for: The Effect of Exogenous Sex Steroids on the Vaginal Microbiota: A Systematic Review
Source: Front Cell Infect Microbiol. 2021 Nov 12;11:732423. doi: 10.3389/fcimb.2021.732423 (PMC8633117; doi:10.3389/fcimb.2021.732423)
Supplement: Supplementary file 1 [file DataSheet_1.pdf]

Supplementary Table 1. Eligibility criteria for inclusion in the systematic review

Eligibility criteria for inclusion in systematic review
Study type
cross-sectional
cohorts
randomised controlled trials
Population type
reproductive-aged women (not currently pregnant)
post-menopausal women
Sex steroid type reported on
hormonal contraceptives stratified by
oestrogen-containing hormonal contraception
progestin-only contraception
menopausal hormone therapy
Molecular methods employed
quantitative polymerase chain reaction (qPCR)
16S microarray
next-generation sequencing
Sanger sequencing
Study outcome: reported on a measure of one/more of the following
composition of the vaginal microbiota (one or more species)
stability of the vaginal microbiota
diversity of the vaginal microbiota

Supplementary Table 2. Bias Assessment Tool

**Selection Bias**	Was the recruited population representative of the general population? E.g. age etc	Yes (low risk) – population was clearly representative	0
		No (high risk) - population was clearly not representative	1
	Were participants randomly allocated? If self-selected treatment, were they consecutively enrolled?	Yes (low risk) – Patients randomly allocated treatment	0
		No (medium risk) – Not randomised, but sequentially enrolled	1
		No (high risk) – Patients self-selected their treatment	2
**Sample Size**	Was the sample size adequate to supporting findings?	Yes (low risk) - Large number of participants sampled (n>100)	0
		Yes (low risk) – Sample size calculations shown and met	0
		No (high risk) – Low number of participants	1
**Measurement Bias**	Were appropriate controls present?	Yes (low risk)- Women not using HC/MHT	0
		Yes (low/medium risk) – Participant baseline specimen as a comparator	1
		No (high risk) – No Controls	2
		No (high risk) - Cu-IUD as a comparator (HC studies ONLY) OR women without PM symptons not on MHT (MHT studies ONLY)	2
	Were the analyses stratified by the HC taken?	Yes (low) – Clearly stratified by oestrogen-containing and progestin-only	0
		N/A MHT study with no stratification needed	0
		No (high) – Unclear whether combined or progestin-only	1
	Adjusted for confounding variables?	Yes (low risk) – Adjusted for confounding variables	0
		No (high risk) – No adjusting for confounding variables	1
**Summary of the overall risk of study bias**		Low Risk	**0-2**
		Moderate Risk	**4-6**
		High Risk	**7-8**

Abbreviation: HC, hormonal contraception; MHT, menopausal hormone therapy; Cu-IUD, copper

Supplementary Table 3. Summary of study populations, design, locations and methods

	Study Population	
**Study Groups**	**HC**	**HRT**
1. Reproductive aged women		
1a. Reproductive aged HIV negative women	20	-
1b. HIV or HIV/HPV positive women	2	-
1c. Women with BV	1	-
1d. Sex workers (no other risk factors)	2	-
2. Post-menopausal women	-	4
**Study Design**		
Cross-sectional	9	1
Longitudinal	16	3
Study duration, madian weeks (range)	31 (1 to 104)	10 (4 to 13)
**Study Region (Defined by WHO)**		
East Asia and Pacific [a]	1	1
Europe and Central Asia	2	0
Latin America and Carribean	2	0
Middle East and North Africa [a]	0	3
North America	8	0
South Asia	0	0
Sub-Saharan Africa	12	0
**Molecular Methods**		
NGS	19	1
qPCR	4	0
Microarray [b]	2	1
Sanger Sequencing [b]	0	3

Abbreviations: HC, hormonal contraception; MHT, menopausal hormone therapy; HIV, Human immunodeficiency virus; HPV, Human papilloma virus; BV, Bacterial vaginosis; NGS, Next-generation sequencing; qPCR, quantitative PCR; DGGE, Denaturing gradient gel Electrophoresis
